# Non-Adherence in Adult Male Patients with Community-Acquired Pneumonia: Relative Forgiveness of Amoxicillin versus Respiratory Fluoroquinolones

**DOI:** 10.3390/antibiotics12050838

**Published:** 2023-05-01

**Authors:** Nerea Carral, John C. Lukas, Oskar Estradé, Nerea Jauregizar, Héctor Morillas, Elena Suárez

**Affiliations:** 1 Department of Pharmacology, School of Medicine and Nursing, University of the Basque Country UPV/EHU, 48940 Leioa, Spain; 2 Drug Modeling & Consulting, Dynakin SL, 48160 Derio, Spain; 3 Department of Analytical Chemistry, Faculty of Science and Technology, University of the Basque Country UPV/EHU, 48080 Bilbao, Spain; 4 Pharmacokinetic, Nanotechnology and Gene Therapy Group (PharmaNanoGene), University of the Basque Country UPV/EHU, 48940 Leioa, Spain; 5 Biocruces Health Research Institute, 48903 Barakaldo, Spain

**Keywords:** amoxicillin, levofloxacin, moxifloxacin, relative forgiveness, non-adherence treatment, simulation, pharmacokinetic-pharmacodynamic

## Abstract

The consequences of non-adherence to treatment (NAT) on antimicrobial efficacy may depend on drug forgiveness—a property that should account for pharmacokinetics (PK) and pharmacodynamics (PD) as well as interindividual variability. In this simulation study, relative forgiveness (RF) in NAT, defined as the probability of a successful PK/PD target (PTA) attained under perfect adherence compared to imperfect adherence, was evaluated for amoxicillin (AMOX) (oral 1000 mg/8 h) and two respiratory fluoroquinolones—levofloxacin (LFX) (oral 750 mg/24 h) and moxifloxacin (MOX) (oral 400 mg/24 h)—in virtual outpatients with community-acquired pneumonia for *S. pneumoniae*. Several NAT scenarios (delay in dose intake and a missed dose) were considered. PK characteristics of virtual patients, including variability in creatinine clearance (70–131 mL/min) and *S. pneumoniae* susceptibility variability associated with geographical location, were simulated in NAT. In this regard, in regions of low MIC delays from 1 h to 7 h or omission of dose ingestion would not have negative consequences on the efficacy of AMOX because of its good RF associated with the AMOX PK and PD properties; RF of LFX 750 mg or MOX 400 mg/24 h regimen vs. AMOX 1000 mg/8 h is one. However, in regions of elevated MIC for *S. pneumoniae* AMOX loses its RF, LFX and MOX vs. AMOX, showing higher RF (>1) depending on the CL_CR_ of patients. These results illustrate the importance of considering the RF of antimicrobial drugs in NAT and provide a framework for further studying its implications for clinical success rates.

## 1. Introduction

Therapeutic adherence to prescribed treatments is one of the pillars of efficient drug use. In this sense, non-adherence to antibiotic therapy (NAT) used in treatment of community-acquired infections has been reported as one of the reasons for therapeutic failure [1,2,3]. Unsurprisingly, patients with NAT could fail to achieve optimal therapeutic drug exposures. Low antimicrobial blood concentrations have also been linked to the development of resistance. Most antimicrobials need to be taken on a strict dose schedule in order to maintain concentrations achieving drug exposure relative to the minimum inhibitory concentration (MIC) for the pathogen [4,5].

Amoxicillin (AMOX), an old antibiotic administered orally at high doses (1000 mg thrice daily regimen), has demonstrated its clinical efficacy and safety in the ambulatory treatment of community-acquired pneumonia without comorbidity (CAP), including COVID infection associated pneumonia, versus other drugs [6,7,8]. A major difficulty is that therapy of CAP must be initiated prior to pathogen identification; thus, empirical treatment with AMOX is usually started immediately [6,7]. Additionally, due to its short half-life AMOX requires frequent administration, typically thrice-daily, complicating outpatient treatment adherence [2]. 

Moreover, the choice of treatment needs to take into account the local prevalence of resistance. *S. pneumoniae* is the main etiological agent for CAP, though its drug sensitivity-resistance pattern varies among regions [9]. In case of resistance to AMOX, respiratory fluoroquinolones (FQ), such as levofloxacin (LFX) and moxifloxacin (MOX), are effective antibiotics [6,7,8], though for clinical use their potential to induce adverse effects should be taken into account [10].

In NAT, it is important to know if the antimicrobial efficacy of the drug, predicted as the probability of reaching pharmacokinetic (PK)/pharmacodynamic (PD) targets, remains. To combat the consequences of NAT, researchers have suggested using so-called ‘‘forgiving’’ drugs. The drug property whereby the therapeutic action duration is longer than the dosing interval is called forgiveness [11,12,13]. 

A forgiveness criterion, such as relative forgiveness (RF), can be used to study the number of times that it is likely to attain a target successfully under perfect adherence compared to imperfect adherence for a given drug or when comparing two drugs under a standard setting of imperfect adherence [12]. Previous research has considered this forgiveness concept in drugs such as warfarin, atorvastatin and omeprazole [12,13].

Forgiveness depends on the PK and PD properties of the drug, as well as the interindividual variability [11,12,14]. During oral administration in CAP, AMOX is a drug with dose-dependent absorption [15], moderate tissue distribution and primarily renal elimination correlated with creatinine clearance (CL_CR_) [16]. LFX and MOX are drugs with favourable PK characteristics, including a high oral bioavailability, volume of distribution and elimination half-life, which allow them to reach adequate concentrations in blood after administration only once per day [17]. Variability in the patient’s CL_CR_, related to factors such as age and serum creatinine (SCr) seems to be responsible for the interindividual variability in systemic clearance for AMOX [16,18] and LFX [19]. In contrast, for MOX, due its elimination mainly by the liver, the plasma clearance varies only with weight but not with age or CL_CR_ [20]. Finally, the antimicrobial PD variability appears to be associated with the geographical variation in pathogen minimum inhibitory concentration (MIC) [9].

The circumstance of how sensitive therapeutic success is measured under imperfect adherence is a critical point in antimicrobial treatment of community-acquired infections. This point has not been studied for the most used antibiotics, such as amoxicillin and respiratory fluoroquinolones. Since it is unethical to investigate NAT in properly designed trials, simulation PK/PD studies were proposed as useful tools to quantify its consequences on drug efficacy [21,22]. 

In this work, we aimed to study the RF of AMOX and the two respiratory FQ—LFX and MOX—in NAT, as well as the RF between these drugs under perfect adherence compared to imperfect adherence scenarios. The role of interindividual PK variability, related to apparent clearance associated with CL_CR_, as well as PD associated with inter-regional differences in MIC, were considered in the study. These characteristics could be greatly importance in considering the forgiveness of drugs in conjunction with the probability of therapeutic success.

## 2. Results

### 2.1. Analysis of the Probability of Target Attainment Related to Clinical Efficacy with AMOX and FQ in NAT

According to the individual patient PK parameters of AMOX and respiratory FQ, LFX and MOX and MIC value for *S. pneumoniae* in susceptible strains (PLS subgroup with low MIC and PHS subgroup with high MIC), simulations on antimicrobial daily regimen yielded different percentage PTA in adherent and non-adherent situations (dose intake delayed for 1–8 h) (Table 1).

In the PHS subgroup, in treatment adherence all virtual patients treated with AMOX and respiratory FQ, LFX and MOX reached PTA ≥ 90%. Maximum delay times of up to 7h and a missed dose are allowed for all drugs, except for the adult subgroup with CL_CR_ = 131 mL/min treated with AMOX, contrary to LFX and MOX.

In the PLS subgroup, PTA ≥ 90% could be reached in all virtual patients except for AMOX in adult populations with CL_CR_ = 131 mL/min in adherent scenari. However, PTA values were <90% and could vary significantly with AMOX and respiratory FQ in the different subgroups in NAT, according CL_CR_ of virtual patients (Table 1). No missing dose is allowed for AMOX (8 h dosing interval), LFX, or MOX (24 h dosing interval).

### 2.2. RF of AMOX and FQ in NAT 

For patients of subgroup PHS, the relative forgiveness (RF) for AMOX, LFX and MOX is one in any NAT scenario, except for AMOX with a missing dose (RF < 1).

**Figure 1:**
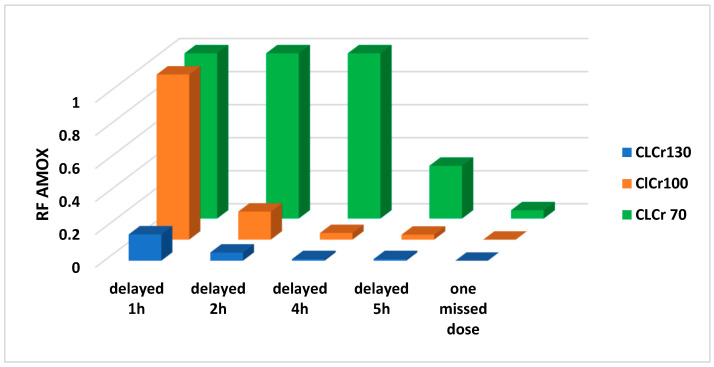
Relative forgiveness (RF) of AMOX 1000 mg administered thrice-daily under perfect adherence compared to imperfect adherence (delayed time 1–5 h in AMOX intake) scenarios in virtual adult patients (range of CL_CR_ = 70–130 mL/min) with community-acquired pneumonia produced by susceptible *S. pneumoniae* strains with high MIC (PLS subgroup).

**Figure 2:**
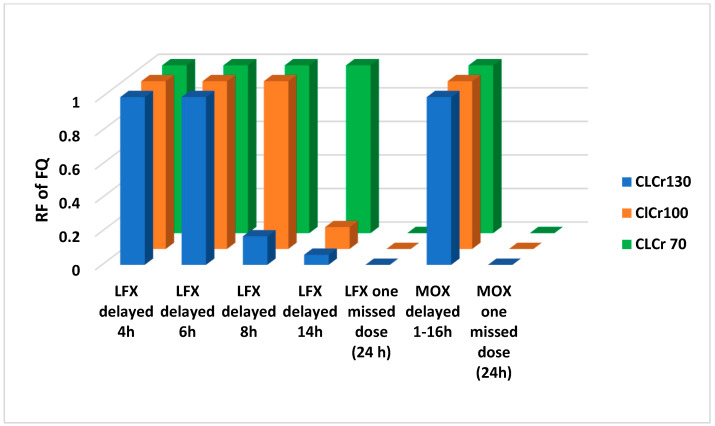
Relative forgiveness (RF) of respiratory Fluoroquinolones (FQ), Levofloxacin 750 mg (LFX) and Moxifloxacin 400 mg (MOX) administered once-daily under perfect adherence compared to imperfect adherence (delayed time 1–16 h and a missed dose) scenarios in virtual adult patients (range of CL_CR_ = 70–130 mL/min) with community-acquired pneumonia produced by susceptible *S. pneumoniae* strains with high MIC (PLS subgroup).

In contrast, for the PLS subgroup delays in the intake of the dose (1–8 h) resulted in different RFs for AMOX and LFX according to CL_CR_ of virtual patients, though not for MOX in NAT (Figure 1 and Figure 2). 

### 2.3. RF of Respiratory Fluoroquinolones versus AMOX in NAT Scenarios

**Figure 3:**
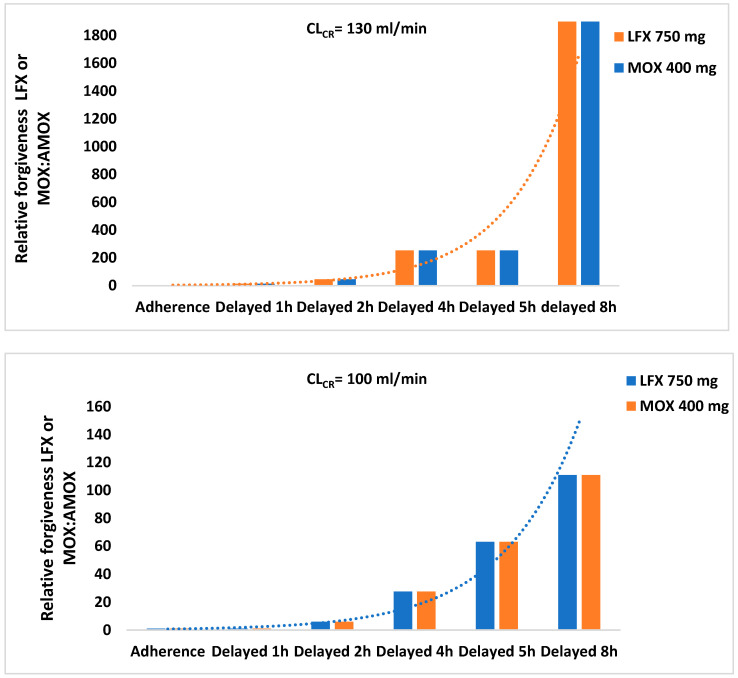

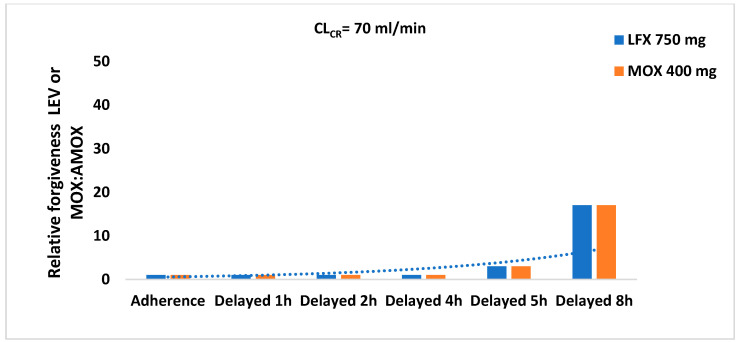
Relative forgiveness of treatment with LFX 750 mg once-daily oral regimen and MOX 400 mg once-daily regimen versus treatment with AMOX 1000 mg thrice daily regimen in scenarios of adherence and non-adherence (delayed 1–8 h) during treatment in virtual adult patients of subgroup CL_CR_ = 70–130 mL/min, with CAP produced by for susceptible *S. pneumonia*e strains with high MIC (subgroup PLS).

In NAT situations for virtual patients of the PHS subgroup, the RF of LFX 750 mg or MOX 400 mg once-daily regimen is one versus the AMOX 1000 mg thrice daily regimen. However, in NAT situations for virtual patients of the PLS subgroup, the RF of LFX 750 mg or MOX 400 mg once-daily regimen versus AMOX 1000 mg thrice-daily regimen in the adult population is higher (RF > 1) and varies depending on the CL_CR_ of patients, as observed in Figure 3.

## 3. Discussion

The starting point for the present simulation study was the absence of information about the impact of NAT on antimicrobial forgiveness in male outpatients with CAP. Mistakes in dosing regimens are common in patients taking antimicrobials, especially when they are administered at short dosing intervals for respiratory tract infections [2]. In the present simulation analysis, adherence and delayed times of up to 8 h (including a missed dose) for AMOX and 24 h for respiratory FQ on the treatment were assumed. As patients typically observe an improvement in their infectious process by the fourth treatment day [23], the above scenarios relate to the increased probability of delaying or skipping a dose under self-medication. Random intermittent alterations in exposure during the entire treatment duration and, consequently, in the probability of reaching PK/PD index predictors of antimicrobial efficacy for AMOX or FQ, as occurred in the non-adherence scenarios simulated in this study, may significantly impact the expected antimicrobial response and the possibility of the appearance of resistance after the interruption [24,25]. 

To analyse the impact of NAT on clinical efficacy after a dosing regimen including all antimicrobials studied, the present simulation study took into account potential interindividual PK variability associated with drug apparent clearance in patients, as well as PD heterogeneity related to the MIC of *S. pneumoniae* susceptible strains and associated with geographical location [9]. Differences in relative forgiveness in NAT could be observed between AMOX, LEV and MOX in clinical settings due their specific PK/PD characteristics. The study demonstrated that, in NAT situations, the local *S. pneumoniae* MIC would play a decisive role in the antimicrobial response. The higher the MIC value (within the MIC range of sensitive strains), the shorter duration of antibiotic concentrations above MIC after dose, with the consequent risk of not reaching the PK/PD target. Secondly, NAT would result in an unintended decrease in drug exposure that could lead to loss of antimicrobial efficacy, measured as percentage PTA, in some outpatient subgroups; its clinical importance is strongly associated with interindividual variability of PK parameters, especially apparent clearance. For AMOX and LEV, the present study reveals differences between male outpatients with a low, intermediate, or high apparent clearance associated to CL_CR_. The subgroup of adult patients in CAP treatment with the highest clearance could be considered at the highest risk of loss of efficacy and poor forgiveness in case of NAT. No differences were observed for MOX on male outpatients with a low, intermediate, or high apparent clearance.

As an illustration, our study showed that, in the PHS subgroup, different delay times in dose ingestion during antimicrobial treatment resulted in lower blood exposure of AMOX compared to adherence scenarios. However, the drug’s PD properties against *S*. *pneumoniae*’s susceptible strains (MIC = 0.064 mg/L) ensure that antibiotic levels are maintained above MIC over 50% of the dosing interval in more than 90% for most patients with CAP. The consequence is that delays from 1 h to 7 h or one omission in dose ingestion related to the patient’s lifestyle would not have negative consequences on the efficacy of AMOX because of its “good forgiveness” associated to the AMOX PK and PD properties, which has been previously proposed for other drugs [11,14,26]. Greater vigilance should be given in NAT for adult male outpatients with high creatinine clearance. In PHS populations, patients with higher clearance of AMOX (20 L/h) would be exposed to a higher risk of loss of antimicrobial efficacy if a dose is missed (8h drug intake delay). 

Interestingly, for PLS populations an important but unanticipated contribution of the study is that the simulations revealed that the PTA ≥ 90% for AMOX could not be reached in the adult population with CL_CR_ = 130 mL/min, even in adherent scenarios (Table 1). A loss of efficacy could be expected when AMOX is prescribed empirically without considering factors such as the CL_CR_ of the individual outpatient with CAP. Similar efficacy problems could be expected in NAT where AMOX is delayed or a missed dose could not be forgiven. A delay of 1–2 h or higher in dose intake can occur frequently within 10 days of treatment, such as by simply adapting the antibiotic intake to breakfast, lunch and dinner [1,2,3]. As shown, these scenarios could not be permitted for adults with CL_CR_ ≥ 100 mL/min (maximum delay allowed of 1 h). 

Consequently, in NAT, AMOX has a good RF in adults with CAP caused by *S. pneumoniae* with low MIC (PHS subgroup), supporting the use of AMOX as first choice for this indication. However, in the group of patients with CAP caused by *S. pneumoniae* with high MIC (PLS subgroup), the lack of adherence to treatment does impair AMOX forgiveness; in this regard, its RF value decreases as the delay interval during treatment increases (Figure 1). Loss of forgiveness depends not only on the high MIC value but also on the pharmacokinetic characteristics of AMOX, the decrease being greater in patients with higher CL_CR_. Therefore, in this type of population the possibility of using other pharmacological alternatives should be considered.

Respiratory FQ, as LFX and MOX, a second alternative in patients with CAP [6,7,8], could be administered in NAT. This study has demonstrated that the RF of LFX and MOX compared with AMOX is far higher and, with a longer delay in drug intake, the forgiveness of FQ versus AMOX increases exponentially. One additional factor is the potential influence of their post-antibiotic effect on actual treatment efficacy in a clinical setting of NAT [27]. Finally, though FQ may be a good therapeutic alternative in CAP scenarios produced by low-susceptible *S. pneumoniae* in patients with NAT, it is not exempt from physician vigilance as missed doses of LFX or MOX result in a drastic decrease in the RF of these antibiotics.

It should not be overlooked that therapeutic decision-making involving FQs needs to consider some serious problems associated with their use, such as the documented risk of side effects affecting muscles, tendons, bones and the nervous system [10]. Clinicians should also take into account the groups of patients in which the highest incidence of adverse effects is expected [10,28,29].

Finally, the methodology used here and in studied sceneries has some limitations that should be discussed. We noted that while, in the case of AMOX, the lack of full population PK studies is striking, the mean behaviour matches that contained in published reports [16,18,30]; the variability was also set to a typical range for all PK parameters. Other relevant pathophysiological alterations not considered in this simulated study were age (children, elderly), weight (obese patients), sex and renal function. The importance of research in females must be considered to learn about the efficient antibiotic effect for CAP in NAT according to gender. Sex is a factor responsible for interindividual variability in CL_CR_ as a consequence of Cl/F for AMOX [16] and LEV [19]. Lastly, though effort is being expended in the PD of the drug, particularly in determining the MIC for *S. pneumoniae* in different geographical regions [9], combined PK and PD knowledge is required to determine the concept of forgiveness for these drugs and apply the concept to the clinical practice of CAP [31].

Our findings suggest that drug-prescribers should consider that, though AMOX is an extensively used and well-known efficacious antibiotic for CAP with a good safety profile, in certain populations associated with geographical regions of low microbial susceptibility the forgiveness could be lost if the patient does not adequately follow the prescribed dosing regimen. In NAT, FQ shows a better forgiveness than AMOX, bearing in mind the benefit/risk ratio in the population. Retrospective clinical observations could be used to corroborate the findings related to adherence to antimicrobial utilisation programs and strategic action plans to reduce the risk of antibiotic resistance.

## 4. Materials and Methods

### 4.1. Imperfect Adherence Scenarios and Virtual Patients

The impact of NAT was studied in virtual male patients with three different monotherapy drug schedules for CAP: AMOX 1000 mg and respiratory FQ as LFX 750 mg and MOX 400 mg [6,7,8]. 

Adult Caucasian male patients aged between 31 and 65 years of age and weighing 70 kg were considered as a virtual outpatient group with CAP. The group was divided into three PK subgroups with increasing creatinine clearance levels (CL_CR_). CL_CR_ was estimated from the Cockcroft–Gault formula for SCr levels between 0.7 to 1.3 mg/dL (variability within normal values of healthy patients), with 70, 100 and 131 mL/min for each subgroup. 

The percentage of *S. pneumoniae* isolates sensitive to antimicrobials and their range of MIC values display large variations depending on geographical region [9]. Thus, two virtual PD subgroups with scenarios of local susceptibilities were chosen according to their antimicrobial susceptibility: for susceptible strains with low MIC, *S. pneumoniae* high antimicrobial susceptibility (PHS subgroup) was chosen, while for susceptible strains with high MIC, *S. pneumoniae* low antimicrobial susceptibility (PLS subgroup) was chosen [32]. Thus, six representative virtual PK/PD patient subgroups were eventually designed for each drug.

In adherence scenarios, AMOX is administered three times daily whilst LFX and MOX are administered orally at once-daily regimens. Drug doses were assumed to be taken at the same time each day for 10 days of CAP treatment. 

Different scenarios of poor adherence were simulated. Several timing errors of increasing duration were considered: dose intake with a delay of 0 h (adherent scenario) or delays from 1 to 7 h after the expected time of dose intake on the 4th treatment day for AMOX or delays from 1 to 24 h after the expected time of dose intake on the 4th treatment day for LFX and MOX. One missing dose after the expected time of dose intake on the 4th treatment day was also simulated.

### 4.2. PK/PD Simulations to Obtain the Probability of Reaching the Target Related to the Antimicrobial Efficacy of AMOX in NAT

A PK model for AMOX was developed using the data from Sjovall et al. [18]; it was used to simulate drug PK after oral dose in virtual subgroups of male outpatients. The PK model was a mixed first- and zero-order absorption one-compartmental model, parameterised with apparent clearance (CL/F) and volume of distribution (Vd/F). A dose-dependent saturation was modelled through the zero-order absorption rate [33,34] (Appendix A). 

AMOX clearance has been previously shown to be creatinine clearance-dependent; thus, it was reparameterised as CL/F = CL_slope x*Cl_CR_/102 [35]. Interindividual coefficients of variation of 20% for the PK parameters were applied and used to generate individual virtual patients (N = 10,000 for each PK subgroup) represented as PK parameter sets (one set for each virtual patient) and simulate their steady-state plasma PK profiles of AMOX. Subgroup populations by CL_CR_ were assumed to arise from the same population PK parameter distributions. PK models and typical PK parameters of AMOX, as well as corresponding values of standard deviations (omega), are summarised in Appendix A. Apparent PK parameters, mean values and standard deviations for AMOX in virtual patients used in the simulation, similar to the values published by De Velde [30], are provided in Supplementary Table S1. An unbound fraction of 82% of AMOX total concentration was considered [36]. Additionally, it has been demonstrated that food intake should not affect AMOX absorption [37]; therefore, food was not included as a confounding factor in our analysis. The MIC values for *S. pneumoniae* from EUCAST [32] were applied and included as variations across regions. Values of 0.064 and 1 mg/L were used by High and Low *S. pneumoniae* antimicrobial susceptibility PHS and PLS subgroups, respectively. 

Time of free antibiotic concentration above the MIC (*f*T_≥MIC_) is the PK/PD parameter used to predict the efficacy of β-lactam antibiotics. In this study, the free drug concentration above the MIC during 50% of the administration time interval (*f*T_50%≥MIC_) was selected as predictor of successful clinical response for AMOX, as previously reported [38]. For each PK/PD subgroup, the *f*T_50%≥MIC_ were estimated with the simulated free drug concentrations and corresponding selected MIC values. The probability of target attainment (% PTA) of *f*T_50%≥MIC_ was calculated at each scenario. The percentage PTA must be ≥90% to predict clinical efficacy [38]. Monte Carlo Simulations were performed with NONMEM v 7.4.3. GraphPad Prism 5.1 was used for graphical purposes.

### 4.3. PK/PD Simulations to Obtain the Probability of Reaching the Target Related the Antimicrobial Efficacy of Respiratory FQ in NAT

The method to study the PTA that predicted the antimicrobial efficacy of respiratory FQ in NAT was previously described by Carral et al. [39] and, according to the reported population PK models for LFX [19] and MOX [20], it was used to simulate drug PK after oral dose in virtual PK subgroups of male outpatients. For LFX, a bicompartmental model with first-order absorption was used. Physiological covariables (age, CL_CR_ and weight) were included as CL predictors in the model [19]. For MOX, a similar bicompartmental model was used where clearance (CL) was a function of the Lean Body Mass (LBM) [20]. PK models and typical PK parameters of LFX and MOX, as well as corresponding values of standard deviations (omega), are summarised in Appendice B and Appendice C and Supplementary Table S2. It should be noted that, in the case of MOX, as its CL does not depend on CL_CR_, only one PK group was studied (CL_CR_ = 70–131 mL/min) instead of three PK subgroups, as conducted for LFX. The area under the free plasma concentration–time curve (0–24 h) (AUC_0–24h_) was calculated according to the relation between dose and CL/F (AUC_0–24h_ = Dose/CL/F). The free area under the free plasma concentration–time curve (*f*AUC_0–24h_) was calculated through multiplying the AUC_0–24h_ with the value of the drug-free fraction. An unbound fraction of 52 for MOX and 69% for LFX total concentration were considered [17].

The MIC values for *S. pneumoniae* from EUCAST [32] were applied and included as variations across regions. MIC values of 0.064 mg/L were used as the High *S. pneumoniae* susceptibility PHS subgroup. MIC values used as the Low *S. pneumoniae* antimicrobial susceptibility PLS subgroup were of 0.25 for MOX and 1 mg/l for AMOX and LFX, respectively.

As the *f*AUC_0–24h_-to-MIC ratio (*f*AUC_0–24h_/MIC ≥ 33.8) was reported to have the strongest correlation with clinical outcomes of fluoroquinolones, it was chosen as a criterion to evaluate treatment efficacy in this study [40]. This PK/PD index was calculated for each patient with the simulated free drug concentrations and corresponding MICs. A total of 10,000 simulations of virtual patients were conducted for each scenario of PK/PD subgroup, which used random samples of the model PK/PD parameter for each drug; the results were averaged over the range of simulation results.

### 4.4. RF of AMOX and Respiratory FQ in NAT

RF—relative forgiveness in NAT—is defined as the probability of a successful PK/PD target (PTA) attained under perfect adherence compared to imperfect adherence. 

To determine the RF of drug in NAT, the criterion proposed by Assawanaski et al. [12] was used. The drugs analysed were AMOX (1000 mg thrice-daily regimen), LFX (750 mg once-daily regimen) and MOX (400 mg once-daily regimen).

A RF of drug under perfect adherence compared to imperfect adherence scenarios is given through the following Equation:

$$\mathrm{RF}=\frac{\mathrm{PTAip}/\left(1-\mathrm{PTAip}\right)}{\mathrm{PTAp}/\left(1-\mathrm{PTAp}\right)}$$

where RF is the relative forgiveness of drug (AMOX or FQ), PTAip is the probability of successful attainment of treatment target under imperfect adherence and PTAp is the probability of successful attainment of treatment target under perfect adherence. RF has the same interpretation as an odds ratio or relative risk. Values of RF close to one indicate that the antimicrobial is forgiving to imperfect, while values close to zero indicate that the antimicrobial is particularly sensitive to imperfect adherence behaviour [12]. 

### 4.5. RF between Respiratory FQ and AMOX in NAT Scenarios

To compare the RF of AMOX with respiratory FQ, LFX and MOX, under a standard setting of imperfect adherence, the criterion proposed by Assawanaski et al. [12] was used:

$$\mathrm{RF}\left(B:A\right)=\frac{\mathrm{PTAip}\left(B\right)/\left(1-\mathrm{PTAip}\left(B\right)\right)}{\mathrm{PTAip}\left(A\right)/\left(1-\mathrm{PTAip}\left(A\right)\right)}$$

In this setting, Drug A was AMOX (1000 mg thrice-daily regimen) and Drug B was LFX (750 mg once-daily regimen) or MOX (400 mg once-daily regimen). PTAip is the probability of successful attainment of drug treatment target under imperfect adherence for different scenarios. 

When RF exceeds the value of 1, it indicates how many times more likely that Drug B is forgiving compared to Drug A, given some pattern of imperfect adherence (i.e., how many times more likely therapeutic success will be achieved with Drug B compared to Drug A).

## 5. Conclusions

The present simulation-based study shows that variability in PK and PD characteristics, such as variability in clearance associated with creatinine clearance and *S. pneumoniae* MIC associated with geographical location would play a decisive role in the AMOX forgiveness in NAT. In regions where, due to high MIC of *S. pneumoniae*, AMOX loses its forgiveness, it may be important to consider other antibiotic alternatives that show greater forgiveness in NAT, such as a LFX 750 mg and MOX 400 mg once-daily regimen. These results illustrate the importance of considering the RF of antimicrobial drugs in NAT and provide a framework for further studying its implications for clinical success rates.

## Figures and Tables

**Table 1:** Probability of attainment of target ratios (% PTA) for *f*T_50%_ ≥MIC for AMOX administered 1000 mg thrice-daily, *f*AUC_0–24h_/MIC ≥ 33.8 for LFX administered 750 mg once-daily and MOX administered 400 mg once-daily in adherence and delayed dose regimen scenarios in subgroups of virtual adult patients according creatinine clearance (CL_CR_). For AMOX, LFX and MOX, MIC was 0.064 mg/L for susceptible strains with low MIC (PHS subgroup). MIC was 1 mg/L for AMOX, LFX and 0.25 mg/L for MOX for susceptible strains with high MIC (PLS subgroup). PTA < 90% are reported in shaded cells. Maximum delay time are reported in red colour.

AMOX	**Subgroups of adults patients according** **CL_CR_ (mL/min)**	**% PTA of *f*T_50%_ ≥ MIC_0.064mg/mL_ in PHS subgroup with delayed dose**				**% PTA of *f*T_50%_ ≥ MIC_1mg/mL_ in PLS subgroup with delayed dose**						
		**Adhe** **rence**	**Delayed time**		**A missed dose (8 h)**	**Adhe** **rence**	**Delayed time**					**A missed dose (8 h)**
			6 h	7 h			1 h	2 h	3 h	4 h	5 h	
	CL_CR_ = 131	100	99	89	66	86	59	30	20	7	3	0
	CL_CR_ = 100	100	100	99	98	99	93	76	52	41	23	6
	CL_CR_ = 70	100	100	100	100	100	100	100	100	93	86	53
LFX	**Subgroups of adults patients according** **CL_CR_ (mL/min)**	**% PTA of *f*AUC_0–24h_/MIC_0.064mg/mL_ in PHS subgroup with delayed dose**				**% PTA of *f*AUC_0–24h_/MIC_1mg/mL_ in PLS subgroup with delayed dose**						
		**Adhe** **rence**	**Delayed time**		**A missed dose (24 h)**	**Adhe** **rence**	**Delayed time**					**A missed dose (24 h)**
			≥12 h				≥6 h		8 h	12 h	14 h	
	CL_CR_ = 131	100	100		100	100	100		76	62	53	0
	CL_CR_ = 100	100	100		100	100	100		100	93	71	0
	CL_CR_ = 70	100	100		100	100	100		100	100	100	0
MOX	**Subgroups of adults patients according** **CL_CR_ (mL/min)**	**% PTA of *f*AUC_0–24h_/MIC_0.064mg/mL_ in PHS subgroup with delayed dose**				**% PTA of *f*AUC_0–24h_/MIC_0.25mg/mL_ in PLS subgroup with delayed dose**						
		**Adhe** **rence**	**Delayed time**		**A missed dose (24 h)**	**Adhe** **rence**	**Delayed time**					**A missed dose (24 h)**
			≥12 h				1 h	4 h	6 h	12 h	14 h	
	CL_CR_ = 70–131	100	100		100	100	100	100	100	100	100	0

## Data Availability

Not applicable.

## Supplementary Materials

The following supporting information n can be downloaded at: https://www.mdpi.com/article/10.3390/antibiotics12050838/s1; Table S1: Simulated pharmacokinetic parameters for amoxicillin (AMOX) in virtual male outpatients, categorized on CL_CR_ subgroups; Table S2: Simulated pharmacokinetic parameters for Levofloxacin (LFX) and Moxifloxacin (MOX) in virtual male outpatients, categorized on CL_CR_ subgroups.

## Appendix A. Amoxicillin: Outline of NONMEM^®^ Control Stream

1. $PROBLEM AMOXICILLIN
2. $INPUT ID TIME DV MDV AMT DOSE CMT RATE
3. $DATA CONTROL.csv IGNORE=I
4. $SUBROUTINES ADVAN2
5. $PK
6. TVKA= THETA(1)
7. KA = THETA(1)*EXP (ETA(1))
8. SIMAGE=45*EXP (ETA(6))
9. SIMWT=70*EXP (ETA(5))
10. CLcr=((140-SIMAGE)*SIMWT)/(Cr*72)
11. TVCL = THETA(2)*(CLcr)/(102)
12. CL/F = TVCL *EXP (ETA(2))
13. TVV = THETA(3)*SIMWT
14. V/F = TVV *EXP (ETA(3))
15. ALAG1 = THETA(4)
16. TVKD = THETA(8)
17. KD = THETA(8)*EXP(ETA(4))
18. F1 = 1-1/(1+THETA(5))
19. DF = 1-DOSE/(KD+DOSE)
20. F2 = 1-F1
21. F2 = F2*DF
22. D2 = THETA(6)
23. ALAG2 = ALAG1+THETA(7)
24. K = CL/V
25. SC = V
26. REPI = IREP
27. $THETA
28. 0.635                                    ;1.KA (1/hr)
29. 15.5                                      ;2.CL (1/hr)
30. 0.33                                      ; 3.V2
31. (0 FIXED)                            ; 4.ALAG1
32. 0.177                                    ; 5.TH5
33. 1.44                                      ; 6.D2
34. 0.190                                    ; 7.ALAG2 (+ALAG1)
35. 1300                                    ; 8.KD
36. $OMEGA ; 004    0.036    0.01    0.04    0.001    0.025
37. $ERROR
38. IPRE = F
39. W = IPRE+.001
40. Y = F+W*EPS(1)
41. $SIGMA   0.4
42. $SIMUL    (657326795)      ONLYSIM

## Appendix B. Levofloxacin: Outline of NONMEM^®^ Control Stream

1. ; PROBLEM LEVOFLOXACIN
2. $INPUT ID TIME AMT DV
3. $DATA LEVO.csv IGNORE=I
4. $SUBROUTINES ADVAN4 TRANS 1
5. $PK
6. SIMIBW=70.11*EXP(ETA(1))
7. SIMAGE=45*EXP(ETA(2))
8. CLcr=((140-SIMAGE)*SIMIBW/(0.9*72)
9. TVCL=THETA(2)-1.486+0.07*CLcr-0.032*SIMAGE
10. CL/F=TVCL*EXP(ETA(3))
11. ;TVCL
12. ;model
13. TVV2=THETA(3)-0.332*SIMAGE+16.51
14. V2/F=YVV2*EXP(ETA(4))
15. S2=V2
16. K=CL/V2
17. TVK23=THETA(4)+0.083
18. K23=TVK23*EXP(ETA(5))
19. TVK32=THETA (5) +SIMAGE*0.005-0.066
20. K32=TVK32*EXP(ETA(6))
21. KA=THETA(6)
22. S2=V2
23. REPI=IREP
24. $ERROR
25. IPRED=F
26. Y=F+F*EPS(1)
27. $THETA
28. 0.7                                  ;THETA(1) Blood creatine mean value
29. 5.94                               ;THETA(2) Constant for prediction of CL
30. 72.096                         ;THETA(3) Regression coefficient for V
31. 0.308                            ;THETA(4) Regression coefficient for K23
32. 0.430                            ;THETA(5) Regression coefficient for K32
33. 2.38                                  ; THETA(6) Ka
34. $OMEGA   0.0003    0.006    0.001    0.04    0.04     0.04
35. $SIGMA                0.029
36. $SIMUL          (657326795)    ONLYSIM

## Appendix C. Moxifloxacin: Outline of NONMEM^®^ Control Stream

1. ; PROBLEM MOXIFLOXACIN
2. $INPUT ID TIME AMT DV
3. $DATA MOXI.csv            IGNORE=I
4. $SUBROUTINES ADVAN4 TRANS4
5. ;TRANS4                CL,V2,Q,V3,KA
6. $PK
7. SIMLBM=57.5*EXP(ETA(1))
8. TVCL/F=THETA(1)*((SIMLBM/60)**0.75))
9. CL/F=TVCL/F*(EXP(ETA(2))
10. TV2/F=THETA(2)*(SIMLBM/(60))
11. V2/F=TV2/F*EXP(ETA(3))
12. TVV3/F=THETA(3)*SIMLBM/(60)
13. V3/F=TVV3/F
14. TVQ=THETA(4)* ((SIMLBM/(60)**0.75)
15. Q=TVQ*EXP(ETA(4))
16. KA=THETA(5)
17. K=CL/V2
18. K23=Q/V2
19. K32= Q/V3
20. S2=V2
21. REPI=IREP
22. $THETA
23. 10          ……………………..;1.CL L/h
24. 131                                        ; 2.V2 L
25. 44.2                                        ; 3.V3 L
26. 4.91                                        ; 4. Q
27. 5.97                                        ; 6.KA 1/h
28. $OMEGA     0.00035      0.045     0.021    0 .25
29. $ERROR
30. IPRED=F
31. Y=F+F*EPS(1)
32. $SIGMA    0.029
33. $SIMUL                        (657326795)           ONLYSIM
