# Supplementary material for: Non-Adherence in Adult Male Patients with Community-Acquired Pneumonia: Relative Forgiveness of Amoxicillin versus Respiratory Fluoroquinolones
Source: Antibiotics (Basel). 2023 May 1;12(5):838. doi: 10.3390/antibiotics12050838 (PMC10215896; doi:10.3390/antibiotics12050838)
Supplement: Supplementary file 1 [file antibiotics-12-00838-s001.zip › antibiotics-2285654-supplementary.pdf]

Supplementary Table S1. Simulated pharmacokinetic parameters for Amoxicillin (AMOX) in virtual male outpatients, categorized on CL_{CR} subgroups. CL_{CR}: creatinine clearance (calculated using the Cockroft-Gault formulae); Vd/F, CL/F and $T_{1/2}$: apparent volume distribution, clearance and elimination half-life parameters respectively. Values are expressed as mean ± standard deviations.

CL_{CR} and PK Parameters	SCr (mg/dL)		
	**0.7**	**0.9**	**1.3**
**CL_{CR} (mL/min)**	131 ± 10	100 ± 8	70 ± 6
**CL/F (L/h)**	20.1 ± 4.0	15.7 ± 3.3	10.8 ± 2.1
**Vd/F (L)**	23.3 ± 3.9	23.3 ± 3.9	23.3 ± 3.9
**$T_{1/2}$ (h)**	0.8 ± 0.2	1 ± 0.2	1.5 ± 0.3

Supplementary Table S2: Simulated pharmacokinetic parameters for Levofloxacin (LFX) and Moxifloxacin (MOX) in virtual male outpatients, categorized on CL_{CR} subgroups. CL_{CR}: creatinine clearance (calculated using the Cockroft-Gault formulae); CL/F: apparent clearance used to AUC calculation. Values are expressed as mean ± standard deviations.

CL_{CR} and PK Parameters	SCr (mg/dL)		
	**0.7**	**0.9**	**1.3**
**CL_{CR} (mL/min)**	131 ± 10	100 ± 8	70 ± 6
**CL/F LFX (L/h)**	14.9 ± 0.6	11 ± 0.9	9.7 ± 0.38
**CL/F MOX (L/h)**	10.1 ± 1.9	10.1 ± 1.9	10.1 ± 1.9
